# Supplementary material for: Hybrid Models and Biological Model Reduction with PyDSTool
Source: PLoS Comput Biol. 2012 Aug 9;8(8):e1002628. doi: 10.1371/journal.pcbi.1002628 (PMC3415397; doi:10.1371/journal.pcbi.1002628)
Supplement: Text S4 — Complete source code for the PyDSTool package (version 0.88.120504). Includes API documentation and help files linking to web pages. This file is identical to the current public release on Sourceforge.net. (ZIP) [file pcbi.1002628.s004.zip › PyDSTool/html/PyDSTool.Generator.ODEsystem'-pysrc.html]

xml version="1.0" encoding="ascii"?


PyDSTool.Generator.ODEsystem'


| Home | Trees | Indices | Help | PyDSTool |
| --- | --- | --- | --- | --- |

Package PyDSTool ::
Package Generator ::
Module ODEsystem'

[hide private]

[frames] | no frames]

# Source Code for Module PyDSTool.Generator.ODEsystem'

```
  1  # ODEsystem base class
 
  2  
 
  3  from allimports import * 
  4  from baseclasses import ctsGen, theGenSpecHelper, auxfn_container 
  5  from PyDSTool.utils import * 
  6  from PyDSTool.common import * 
  7  from PyDSTool.Variable import Variable, iscontinuous 
  8  from PyDSTool.Trajectory import Trajectory 
  9  from PyDSTool.Points import Pointset 
 10  from PyDSTool.Interval import uncertain 
 11  
 
 12  # Other imports
 
 13  from numpy import Inf, NaN, isfinite, sometrue, alltrue, array, arange, \
 
 14       zeros, float64, int32, transpose, shape 
 15  import math, random 
 16  import types 
 17  from copy import copy, deepcopy 
 18  try: 
 19      # use pscyo JIT byte-compiler optimization, if available
 
 20      import psyco 
 21      HAVE_PSYCO = True 
 22  except ImportError: 
 23      HAVE_PSYCO = False 
 24  
 
 25  
 
 26  
 


27 -class ODEsystem(ctsGen):


28      """Abstract class for ODE system solvers.""" 
 29      _validKeys = ['globalt0', 'xdomain', 'tdata', 'tdomain', 'checklevel',
 
 30                    'ics', 'pars', 'algparams', 'inputs', 'pdomain', 'abseps',
 
 31                    'inputs_t0'] 
 32      _needKeys = ctsGen._needKeys + ['varspecs'] 
 33      _optionalKeys = ctsGen._optionalKeys + ['tdomain', 'xdomain', 'xtype',
 
 34                                              'inputs', 'tdata',
 
 35                            'ics', 'events', 'compiler', 'enforcebounds',
 
 36                            'activatedbounds', 'checklevel', 'algparams',
 
 37                            'auxvars', 'vars', 'pars', 'fnspecs', 'pdomain',
 
 38                            'reuseterms', 'vfcodeinsert_start',
 
 39                            'vfcodeinsert_end', 'ignorespecial'] 
 40  
 


41 -    def __init__(self, kw):


42          ctsGen.__init__(self, kw) 
 43          self.diagnostics._errmessages[E_COMPUTFAIL] = 'Integration failed' 
 44          # user auxiliary function interface
 
 45          self.auxfns = auxfn_container(self) 
 46          dispatch_list = ['varspecs', 'tdomain', 'tdata', 'inputs',
 
 47                          'ics', 'allvars', 'xtype', 'xdomain',
 
 48                          'reuseterms', 'algparams', 'pars', 'pdomain',
 
 49                          'fnspecs', 'target', 'vfcodeinserts', 'ignorespecial'] 
 50          # process keys and build func spec
 
 51          self.funcspec = RHSfuncSpec(self._kw_process_dispatch(dispatch_list,
 
 52                                                                kw)) 
 53          self.indepvartype = float 
 54          for v in self.inputs.values(): 
 55              if not iscontinuous(v): 
 56                  raise ValueError("External inputs for ODE system must be "
 
 57                         "continuously defined") 
 58          self._kw_process_events(kw) 
 59          self.checkArgs(kw) 
 60          tindepdomain = Interval('t_domain', self.indepvartype, self.tdomain,
 
 61                                  self._abseps) 
 62          tdepdomain = Interval('t', self.indepvartype, self.tdata, self._abseps) 
 63          self.indepvariable = Variable(listid, tindepdomain, tdepdomain, 't') 
 64          self._register(self.indepvariable) 
 65          for xname in self.funcspec.vars + self.funcspec.auxvars: 
 66              # Add a temporary dependent variable domain, for validation testing
 
 67              # during integration
 
 68              self.variables[xname] = Variable(indepdomain=tdepdomain,
 
 69                                               depdomain=Interval(xname,
 
 70                                                            self.xtype[xname],
 
 71                                                            self.xdomain[xname],
 
 72                                                            self._abseps)) 
 73          self._register(self.variables) 
 74          self._generate_ixmaps() 
 75          # Introduce any python-specified code to the local namespace
 
 76          self.addMethods() 
 77          # all registration completed
 
 78          self.validateSpec()

 79  
 
 80  
 


81 -    def prepDirection(self, dirn):


82          """Common pre-integration tasks go here""" 
 83          if dirn in ['f', 'forward', 1]: 
 84              self._dircode = 1 
 85              continue_integ = False 
 86          elif dirn in ['b', 'backward', -1]: 
 87              self._dircode = -1 
 88              continue_integ = False 
 89          elif dirn in ['c', 'continue', 0]: 
 90              if self.defined and self._solver is not None: 
 91                  continue_integ = True 
 92              else: 
 93                  # just treat as initial forwards integration
 
 94                  continue_integ = False 
 95                  self._dircode = 1 
 96          else: 
 97              raise ValueError('Invalid direction code in argument') 
 98          return continue_integ

 99  
 
100  
 


101 -    def addMethods(self, usePsyco=False):


102          """Add Python-specific functions to this object's methods,
 
103          accelerating them with psyco, if it is available.""" 
104  
 
105          # Add the auxiliary function specs to this Generator's namespace
 
106          for auxfnname in self.funcspec._pyauxfns: 
107              fninfo = self.funcspec._pyauxfns[auxfnname] 
108              if not hasattr(self, fninfo[1]): 
109                  # user-defined auxiliary functions
 
110                  # (built-ins are provided explicitly)
 
111                  try: 
112                      exec fninfo[0] 
113                  except: 
114                      print 'Error in supplied auxiliary function code' 
115                  self._funcreg[fninfo[1]] = ('self', fninfo[0]) 
116                  setattr(self, fninfo[1], types.MethodType(locals()[fninfo[1]],
 
117                                                             self,
 
118                                                             self.__class__)) 
119                  # user auxiliary function interface wrapper
 
120                  try: 
121                      uafi_code = self.funcspec._user_auxfn_interface[auxfnname] 
122                      try: 
123                          exec uafi_code 
124                      except: 
125                          print 'Error in auxiliary function wrapper' 
126                          raise 
127                      setattr(self.auxfns, auxfnname,
 
128                              types.MethodType(locals()[auxfnname], self.auxfns,
 
129                                           auxfn_container)) 
130                      self._funcreg[auxfnname] = ('', uafi_code) 
131                  except KeyError: 
132                      # not a user-defined aux fn
 
133                      pass 
134              # bind all the auxfns here
 
135              if HAVE_PSYCO and usePsyco: 
136                  psyco.bind(getattr(self, fninfo[1])) 
137                  try: 
138                      psyco.bind(self.auxfns[auxfnname]) 
139                  except KeyError: 
140                      # not a user-defined aux fn
 
141                      pass 
142          # Add the spec function to this Generator's namespace if
 
143          # target language is python (otherwise integrator exposes it anyway)
 
144          if self.funcspec.targetlang == 'python': 
145              fninfo = self.funcspec.spec 
146              try: 
147                  exec fninfo[0] 
148              except: 
149                  print 'Error in supplied functional specification code' 
150                  raise 
151              self._funcreg[fninfo[1]] = ('self', fninfo[0]) 
152              setattr(self, fninfo[1], types.MethodType(locals()[fninfo[1]],
 
153                                                             self,
 
154                                                             self.__class__)) 
155              if HAVE_PSYCO and usePsyco: 
156                  psyco.bind(getattr(self, fninfo[1])) 
157              # Add the auxiliary spec function (if present) to this
 
158              # Generator's namespace
 
159              if self.funcspec.auxspec != '': 
160                  fninfo = self.funcspec.auxspec 
161                  try: 
162                      exec fninfo[0] 
163                  except: 
164                      print 'Error in supplied auxiliary variable code' 
165                      raise 
166                  self._funcreg[fninfo[1]] = ('self', fninfo[0]) 
167                  setattr(self, fninfo[1], types.MethodType(locals()[fninfo[1]],
 
168                                                             self,
 
169                                                             self.__class__)) 
170                  if HAVE_PSYCO and usePsyco: 
171                      psyco.bind(getattr(self, fninfo[1]))

172  
 
173  
 


174 -    def haveJacobian(self):


175          """Report whether ODE system has an explicit user-specified Jacobian
 
176          associated with it.""" 
177          return 'Jacobian' in self.funcspec.auxfns

178  
 
179  
 


180 -    def haveJacobian_pars(self):


181          """Report whether ODE system has an explicit user-specified Jacobian
 
182          with respect to pars associated with it.""" 
183          return 'Jacobian_pars' in self.funcspec.auxfns

184  
 
185  
 


186 -    def haveMass(self):


187          """Report whether ODE system has an explicit user-specified mass matrix
 
188          associated with it.""" 
189          return 'massMatrix' in self.funcspec.auxfns

190  
 
191  
 


192 -    def checkInitialConditions(self, checkauxvars=False):


193          for xname, val in self.initialconditions.iteritems(): 
194              if xname not in self.funcspec.vars and not checkauxvars: 
195                  # auxvars do not need initial conditions unless
 
196                  # explicitly requested (e.g. for user call to RHS
 
197                  # function of C code vector field before trajectory
 
198                  # has been computed)
 
199                  continue 
200              try: 
201                  if not isfinite(val): 
202                      raise ValueError("Initial condition for "+xname+" has been "
 
203                                      "incorrectly initialized") 
204              except TypeError: 
205                  print "Found: ", val 
206                  print "of type: ", type(val) 
207                  raise TypeError("Invalid type for %s`s initial"%xname \
 
208                                  + "condition value") 
209              if not self.contains(self.variables[xname].depdomain,
 
210                                   val, self.checklevel): 
211                  print "Bounds: ", self.variables[xname].depdomain.get() 
212                  print "Variable value: ", val 
213                  raise ValueError("Initial condition for "+xname+" has been "
 
214                                     "set outside of prescribed bounds")

215  
 
216  
 


217 -    def set(self, **kw):


218          """Set ODE system parameters""" 
219          if remain(kw.keys(), self._validKeys) != []: 
220              raise KeyError("Invalid keys in argument") 
221          if 'globalt0' in kw: 
222              # pass up to generic treatment for this
 
223              ctsGen.set(self, globalt0=kw['globalt0']) 
224              # set this True so that can adjust the input time arrays
 
225              # to new globalt0
 
226              self._extInputsChanged = True 
227          if 'checklevel' in kw: 
228              # pass up to generic treatment for this
 
229              ctsGen.set(self, checklevel=kw['checklevel']) 
230          if 'abseps' in kw: 
231              # pass up to generic treatment for this
 
232              ctsGen.set(self, abseps=kw['abseps']) 
233          # optional keys for this call are ['pars', 'tdomain', 'ics',
 
234          #   'algparams', 'tdata', 'xdomain', 'pdomain', 'inputs']
 
235          if 'ics' in kw: 
236              for k_temp, v in kw['ics'].iteritems(): 
237                  k = self._FScompatibleNames(k_temp) 
238                  if k in self.funcspec.vars+self.funcspec.auxvars: 
239                      self._xdatadict[k] = ensurefloat(v) 
240                  else: 
241                      raise ValueError('Illegal variable name %s'%k) 
242              self.initialconditions.update(self._xdatadict) 
243          tchange = False 
244          if 'tdata' in kw: 
245              self.tdata = kw['tdata'] 
246              tchange = True 
247          if 'tdomain' in kw: 
248              self.tdomain = kw['tdomain'] 
249              self.indepvariable.indepdomain.set(self.tdomain) 
250              tchange = True 
251          if tchange: 
252              if self.tdomain[0] > self.tdata[0]: 
253                  if self.indepvariable.indepdomain.contains(self.tdata[0]) == uncertain: 
254                      self.diagnostics.warnings.append((W_UNCERTVAL,
 
255                                                        (self.tdata[0],self.tdomain))) 
256                  else: 
257                      print 'tdata cannot be specified below smallest '\
 
258                            'value in tdomain\n (possibly due to uncertain bounding).'\
 
259                            ' It has been automatically adjusted from\n ', \
 
260                            self.tdata[0], 'to', self.tdomain[0], '(difference of', \
 
261                            self.tdomain[0]-self.tdata[0], ')' 
262                      if self._modeltag: 
263                          print 'Try reducing step size in model.' 
264                  self.tdata[0] = self.tdomain[0] 
265              if self.tdomain[1] < self.tdata[1]: 
266                  if self.indepvariable.indepdomain.contains(self.tdata[1]) == uncertain: 
267                      self.diagnostics.warnings.append((W_UNCERTVAL,
 
268                                                        (self.tdata[1],self.tdomain))) 
269                  else: 
270                      print 'tdata cannot be specified above largest '\
 
271                            'value in tdomain\n (possibly due to uncertain bounding).'\
 
272                            ' It has been automatically adjusted from\n ', \
 
273                            self.tdomain[1], 'to', \
 
274                            self.tdomain[1], '(difference of', \
 
275                            self.tdata[1]-self.tdomain[1], ')' 
276                      if self._modeltag: 
277                          print 'Try reducing step size in model.' 
278                  self.tdata[1] = self.tdomain[1] 
279              self.indepvariable.depdomain.set(self.tdata) 
280          if 'xdomain' in kw: 
281              for k_temp, v in kw['xdomain'].iteritems(): 
282                  k = self._FScompatibleNames(k_temp) 
283                  if k in self.funcspec.vars+self.funcspec.auxvars: 
284                      if isinstance(v, _seq_types): 
285                          assert len(v) == 2, \
 
286                                 "Invalid size of domain specification for "+k 
287                          if v[0] >= v[1]: 
288                              raise PyDSTool_ValueError('xdomain values must be'
 
289                                                        'in order of increasing '
 
290                                                        'size') 
291                          else: 
292                              self.xdomain[k] = copy(v) 
293                      elif isinstance(v, _num_types): 
294                          self.xdomain[k] = [v, v] 
295                      else: 
296                          raise PyDSTool_TypeError('Invalid type for xdomain spec'
 
297                                                   ' '+k) 
298                  else: 
299                      raise ValueError('Illegal variable name') 
300                  try: 
301                      self.variables[k].depdomain.set(v) 
302                  except TypeError: 
303                      raise TypeError('xdomain must be a dictionary of variable'
 
304                                        ' names -> valid interval 2-tuples or '
 
305                                        'singletons') 
306                  try: 
307                      evs = self.eventstruct.events.values() 
308                  except AttributeError: 
309                      evs = [] 
310                  for ev in evs: 
311                      ev.xdomain[k] = self.xdomain[k] 
312          if 'pdomain' in kw: 
313              for k_temp, v in kw['pdomain'].iteritems(): 
314                  k = self._FScompatibleNames(k_temp) 
315                  if k in self.funcspec.pars: 
316                      if isinstance(v, _seq_types): 
317                          assert len(v) == 2, \
 
318                                 "Invalid size of domain specification for "+k 
319                          if v[0] >= v[1]: 
320                              raise PyDSTool_ValueError('pdomain values must be'
 
321                                                        'in order of increasing '
 
322                                                        'size') 
323                          else: 
324                              self.pdomain[k] = copy(v) 
325                      elif isinstance(v, _num_types): 
326                          self.pdomain[k] = [v, v] 
327                      else: 
328                          raise PyDSTool_TypeError('Invalid type for pdomain spec'
 
329                                                   ' '+k) 
330                  else: 
331                      raise ValueError('Illegal parameter name') 
332                  try: 
333                      self.parameterDomains[k].depdomain.set(v) 
334                  except TypeError: 
335                      raise TypeError('xdomain must be a dictionary of parameter'
 
336                                        ' names -> valid interval 2-tuples or '
 
337                                        'singletons') 
338                  try: 
339                      evs = self.eventstruct.events.values() 
340                  except AttributeError: 
341                      evs = [] 
342                  for ev in evs: 
343                      ev.pdomain[k] = self.pdomain[k] 
344          if 'pars' in kw: 
345              for k_temp, v in kw['pars'].iteritems(): 
346                  k = self._FScompatibleNames(k_temp) 
347                  if k in self.pars: 
348                      cval = self.parameterDomains[k].contains(v) 
349                      if self.checklevel < 3: 
350                          if cval is not notcontained: 
351                              self.pars[k] = ensurefloat(v) 
352                              if cval is uncertain and self.checklevel == 2: 
353                                  print 'Warning: Parameter %s: value at bound'%str(k) 
354                          else: 
355                              raise PyDSTool_ValueError('Parameter %s: value out of bounds'%str(k)) 
356                      else: 
357                          if cval is contained: 
358                              self.pars[k] = ensurefloat(v) 
359                          elif cval is uncertain: 
360                              raise PyDSTool_UncertainValueError('Parameter %s: value at bound'%str(k)) 
361                          else: 
362                              raise PyDSTool_ValueError('Parameter %s: value out of bounds'%str(k)) 
363                  else: 
364                      raise PyDSTool_ValueError('Illegal parameter name '+str(k)) 
365          if 'algparams' in kw: 
366              for k, v in kw['algparams'].iteritems(): 
367                  self.algparams[k] = v 
368                  if k in ('eventActive', 'eventTol', 'eventDelay', 'eventDir', 'eventInt', 'eventTerm',
 
369                           'maxbisect'): 
370                      raise ValueError("Use appropriate setX method in Generator.eventstruct") 
371          if 'inputs' in kw: 
372              assert self.inputs, ('Cannot provide inputs after '
 
373                                  'initialization without them') 
374              inputs = copy(kw['inputs']) 
375              _inputs = {} 
376              if isinstance(inputs, Trajectory): 
377                  # extract the variables
 
378                  _inputs = self._FScompatibleNames(inputs.variables) 
379              elif isinstance(inputs, Variable): 
380                  _inputs = {self._FScompatibleNames(inputs.name): inputs} 
381              elif isinstance(inputs, Pointset): 
382                  # turn into Variables
 
383                  for n in inputs.coordnames: 
384                      x_array = inputs[n] 
385                      nFS = self._FScompatibleNames(n) 
386                      _input[nFS] = Variable(interp1d(inputs.indepvararray,
 
387                                                      x_array), 't',
 
388                                             Interval(nFS, float, extent(x_array),
 
389                                                      abseps=self._abseps),
 
390                                             name=n) 
391              elif isinstance(inputs, dict): 
392                  _inputs = self._FScompatibleNames(inputs) 
393                  # ensure values are Variables
 
394                  for v in _inputs.values(): 
395                      if not isinstance(v, Variable): 
396                          raise TypeError("Invalid specification of inputs") 
397              else: 
398                  raise TypeError("Invalid specification of inputs") 
399              for v in _inputs.values(): 
400                  if not iscontinuous(v): 
401                      raise ValueError("External inputs for ODE system must be "
 
402                                       "continously defined") 
403              if _inputs: 
404                  for i in _inputs: 
405                      assert i in self.inputs, 'Incorrect input name provided' 
406                      self.inputs[i] = _inputs[i] 
407                  # re-calc inputs ixmaps
 
408                  self._generate_ixmaps('inputs') 
409              self._extInputsChanged = True 
410          if 'inputs_t0' in kw: 
411              assert self.inputs, ('Cannot provide inputs after '
 
412                                  'initialization without them') 
413              inputs_t0 = self._FScompatibleNames(kw['inputs_t0']) 
414              for iname, t0 in inputs_t0.items(): 
415                  self.inputs[iname]._internal_t_offset = t0 
416              self._extInputsChanged = True

417  
 
418  
 


419 -    def compute(self, dirn):


420          """This is an abstract class.""" 
421  
 
422          # Don't call this from a concrete sub-class
 
423          if self.__class__ is ODEsystem: 
424              raise NotImplementedError('ODEsystem is an abstract class. '
 
425                                'Use a concrete sub-class of ODEsystem.') 
426          else: 
427              raise NotImplementedError("This Generator does not support "
 
428                                "calls to compute()")

429  
 


430 -    def cleanupMemory(self):


431          """Clean up memory usage from past runs of a solver that is interfaced through
 
432          a dynamic link library. This will prevent the 'continue' integration option from
 
433          being accessible and will delete other data about the last integration run.""" 
434          if hasattr(gen, '_solver'): 
435              # clean up memory usage after calculations in Dopri and Radau, or other
 
436              # solvers that we have interfaced to
 
437              try: 
438                  gen._solver.CleanupEvents() 
439                  gen._solver.CleanupInteg() 
440              except AttributeError: 
441                  # incompatible solver, so no need to worry
 
442                  pass

443  
 


444 -    def validateICs(self):


445          assert hasattr(self, 'initialconditions') 
446          if remain(self.initialconditions.keys(),
 
447                        self.variables.keys()) != []: 
448              print "IC names defined:", self.initialconditions.keys() 
449              print "Varnames defined:", self.variables.keys() 
450              raise ValueError("Mismatch between initial condition and variable "
 
451                               "names") 
452          for v in self.funcspec.vars: 
453              try: 
454                  assert self.initialconditions[v] is not NaN, ('Must specify'
 
455                                      ' initial conditions for all variables') 
456              except KeyError: 
457                  raise KeyError("Variable name "+v+" not found in initial"
 
458                                   " conditions")

459  
 
460  
 


461 -    def Rhs(self, t, xdict, pdict=None, asarray=False):


462          # Don't call this from a concrete sub-class
 
463          if self.__class__ is ODEsystem: 
464              raise NotImplementedError('ODEsystem is an abstract class. '
 
465                                'Use a concrete sub-class of ODEsystem.') 
466          else: 
467              raise NotImplementedError("This Generator does not support "
 
468                                "calls to Rhs()")

469  
 
470  
 


471 -    def Jacobian(self, t, xdict, pdict=None, asarray=False):


472          # Don't call this from a concrete sub-class
 
473          if self.__class__ is ODEsystem: 
474              raise NotImplementedError('ODEsystem is an abstract class. '
 
475                                'Use a concrete sub-class of ODEsystem.') 
476          else: 
477              raise NotImplementedError("This Generator does not support "
 
478                                "calls to Jacobian()")

479  
 
480  
 


481 -    def JacobianP(self, t, xdict, pdict=None, asarray=False):


482          # Don't call this from a concrete sub-class
 
483          if self.__class__ is ODEsystem: 
484              raise NotImplementedError('ODEsystem is an abstract class. '
 
485                                'Use a concrete sub-class of ODEsystem.') 
486          else: 
487              raise NotImplementedError("This Generator does not support "
 
488                                "calls to JacobianP()")

489  
 
490  
 


491 -    def AuxVars(self, t, xdict, pdict=None, asarray=False):


492          # Don't call this from a concrete sub-class
 
493          if self.__class__ is ODEsystem: 
494              raise NotImplementedError('ODEsystem is an abstract class. '
 
495                                'Use a concrete sub-class of ODEsystem.') 
496          else: 
497              raise NotImplementedError("This Generator does not support "
 
498                                "calls to AuxFunc()")

499  
 
500  
 
501      # Methods for pickling protocol
 


502 -    def __getstate__(self):


503          d = copy(self.__dict__) 
504          for fname, finfo in self._funcreg.iteritems(): 
505              try: 
506                  del d[fname] 
507              except KeyError: 
508                  pass 
509          # delete user auxiliary function interface
 
510          try: 
511              del d['auxfns'] 
512          except KeyError: 
513              pass 
514          # can't pickle module objects, so ensure that _solver deleted:
 
515          # Dopri and Radau don't have _solver in funcreg
 
516          try: 
517              del d['_solver'] 
518          except KeyError: 
519              pass 
520          return d

521  
 
522  
 


523 -    def __setstate__(self, state):


524          self.__dict__.update(state) 
525          self._solver = None 
526          if self._funcreg != {}: 
527              self.auxfns = auxfn_container(self) 
528              self.addMethods()

529  
 
530  
 


531 -    def __del__(self):


532          ctsGen.__del__(self)

533
```

  


| Home | Trees | Indices | Help | PyDSTool |
| --- | --- | --- | --- | --- |

|  |  |
| --- | --- |
| Generated by Epydoc 3.0.1 on Fri May 4 15:24:15 2012 | http://epydoc.sourceforge.net |
